# Thriving Beyond Resilience Despite Stress: A Psychometric Evaluation of the Newly Developed Teacher Stress Scale and Teacher Thriving Scale

**DOI:** 10.3389/fpsyg.2022.862342

**Published:** 2022-05-24

**Authors:** Jennifer J. Chen, Zijia Li, Wilson Rodrigues, Samantha Kaufman

**Affiliations:** ^1^Program of Early Childhood and Family Studies, School of Curriculum and Teaching, College of Education, Kean University, Union, NJ, United States; ^2^National Institute for Early Education Research, Graduate School of Education, Rutgers, The State University of New Jersey, New Brunswick, NJ, United States

**Keywords:** early childhood teachers, stress coping, teacher stress, Teacher Stress Scale, teacher thriving, Teacher Thriving Scale

## Abstract

Building on theoretical and empirical insights and applying the thriving theory as the conceptual framework, the authors developed two new teacher-specific scales, namely the Teacher Stress Scale (TSS) and the Teacher Thriving Scale (TTS). The goal of this investigation was to evaluate the psychometric properties of these two scales. Data were collected through an online questionnaire administered to a national sample of 122 participating early childhood teachers (ages 22–72 years, *M* = 41.01) teaching in preschool through third grade in 26 states of the United States during the 2020–2021 school year amidst COVID-19. This study revealed some important psychometric results. First, with respect to their internal structures, both the TSS and the TTS appeared to be best represented as bifactorial and trifactorial, respectively. Specifically, the TSS comprised two constructs: (1) Inadequate School-based Support, and (2) Teaching-related Demands; and the TTS encompassed three constructs: (1) Adaptability and Flexibility, (2) Personal Strengths and Professional Growth, and (3) Positive Mindset. Second, the negative correlation between the TSS and the TTS provided discriminant evidence for each other’s construct validity, while the positive correlations between the TTS and six conceptually cognate constructs (Stress Resilience, Resilience Coping, Coping Efficacy, Teaching Satisfaction, Emotional Support, and Gratitude) demonstrated convergent evidence for construct validity for the TTS. Third, both the overall TSS and the overall TTS as well as their subscales exhibited good internal consistency reliability. Fourth, both the overall TSS and the overall TTS also demonstrated test–retest reliability.

## Introduction

Since early 2020, the unprecedented COVID-19 pandemic has upended humanity globally. In the education realm, it has disrupted the traditional rhythm of the teaching and learning process. In response to the lingering pandemic, during the 2020–2021 school year throughout the United States, many school districts sought to balance between keeping everyone safe and providing an optimal learning experience for students by oscillating among three unconventional instructional modalities: (1) virtual/remote, (2) in-person (following federal and state health and safety protocols), and (3) a hybrid of the two. The novelty and variability of this educational process might have portended new trials and tribulations for educators, especially early childhood teachers caring for and educating young children with unique developmental and learning needs. Early childhood teachers in the United States refer to those teaching young children (ages birth-8 years/third grade) (National Association for the Education of Young Children [NAEYC], 2022).

Even before COVID-19, early childhood teachers in the United States had already been enduring high levels of stress (e.g., Whitaker et al., 2015; Roberts et al., 2016; Tebben et al., 2021). During COVID-19, the unconventional instructional modalities nationwide have imposed new additional teaching-related demands and challenges on early childhood teachers, thereby exacerbating their stress levels (e.g., Bassok et al., 2020; Tarrant and Nagasawa, 2020; Randall et al., 2021; Chen, 2022). As of March 2022, it has been 2 years since the advent of COVID-19 in the United States. Yet, only scant empirical efforts (e.g., Bassok et al., 2020; Tarrant and Nagasawa, 2020; Randall et al., 2021; Chen, 2022) have examined early childhood teachers’ experience of teacher stress during this pandemic. It behooves researchers to examine stress and stress coping among early childhood teachers teaching during COVID-19 to better inform the provision of support to these teachers.

The current literature on teachers’ adaptive stress coping focuses largely on stress resilience (e.g., Gu and Day, 2007; Day and Gu, 2014; Day and Hong, 2016; Whitebook et al., 2018). Although resilience is a constructive response to adversity, the thriving theory (O’Leary and Ickovics, 1995; Carver, 1998) suggests that thriving is an even more optimal coping mechanism because it surpasses resilience to embrace challenges as opportunities for positive change that will lead to personal and professional growth. Understandably, because of the conceptual affinity between resilience and thriving, previous research on teacher stress coping tends to lump the two together in a shared discourse (e.g., Sumsion, 2003, 2004; Howard and Johnson, 2004; Gu and Day, 2007; Malloy and Allen, 2007). Thus, it is unclear if and how teachers thrive in the face of teaching-related stress. To contribute clarity to this area of inquiry, we administered our newly developed Teacher Stress Scale (TSS) and Teacher Thriving Scale (TTS) to a national sample of early childhood teachers in the United States to gauge their capacity to potentially thrive despite stress. It was expected that the findings would provide guidance for support practices that could enhance early childhood teachers’ psychological wellbeing.

## Definitions of Teacher Stress

Teaching is among the most stressful professions (Johnson et al., 2005). Recognizing the prevalence of stress experienced by teachers, Kyriacou and Sutcliffe (1977) accorded this phenomenon an occupation-specific term, “teacher stress.” Teacher stress has since been defined variously by scholars. For instance, Kyriacou (2001) defined teacher stress “as the experience by a teacher of unpleasant, negative emotions, such as anger, anxiety, tension, frustration or depression, resulting from some aspect of their work as a teacher” (p. 28). Prilleltensky et al. (2016) further operationalized teacher stress as manifested specifically in “an imbalance between risk and protective factors…When risk factors exceed protective factors, teacher ability to cope with adversity is inhibited, likely resulting in stress and pernicious consequences” (p. 104). Essentially, teacher stress is viewed as an adverse psychological response to a particular situation, reflecting a syndrome of emotional turmoil (Kyriacou, 2001) or inadequate protective resources (Prilleltensky et al., 2016). The corollary is that combating teacher stress would require teachers to restore their emotional balance and/or acquire more protective than risk factors.

## The Prevalence of Teacher Stress Among Early Childhood Teachers in the United States

Across the United States, teaching is one of the most critical sectors in society. Yet, it is also among the most stressful occupations. Data from the 2013 Gallup-Healthways Well-Being Index revealed that nearly half of the K-12 teachers (46%) reported experiencing high levels of daily stress during the school year, paralleling other exceedingly demanding professions, such as nurses (46%) and doctors (45%) (Gallup, 2014). While teacher stress in the United States is pervasive in general, stress experienced by early childhood educators is particularly prevalent (e.g., Whitaker et al., 2015; Roberts et al., 2016; Tebben et al., 2021). For example, in their large-scale survey-based study of 2,199 preschool educators working with young children in 66 Head Start programs in Pennsylvania, Whitaker et al. (2013) found that these educators experienced significantly poorer levels of mental health, with 23% of them reporting diagnosed depression compared to 17.6% of two national samples of peers with similar sociodemographic characteristics.

Teacher stress among early childhood teachers is a pervasive yet complex phenomenon. Its antecedents and correlates can be multifarious, including a lack of support system and poor work conditions (g., Jeon and Ardeleanu, 2020; Clayback and Williford, 2021; Tebben et al., 2021), and additional teaching challenges during COVID-19 (Nagasawa and Tarrant, 2020; Tarrant and Nagasawa, 2020). For instance, in their secondary analysis of a sample of 427 preschool teachers in the United States, Clayback and Williford (2021) found that both individual and environmental factors (e.g., a lack of administrative support) predicted teacher stress. Similarly, in their study of 1,129 preschool-aged classroom teachers across the United States, Jeon and Ardeleanu (2020) found that early childhood teachers who perceived their work climate more negatively (i.e., poorer work situations, less support from the students’ families, more incidents of children’s challenging behaviors) reported more teacher stress.

While the aforementioned research has identified antecedents of teacher stress, other empirical studies have revealed consequences of teacher stress among early childhood teachers in the United States. These consequences include poorer teacher efficacy in supporting the learning and development of young children (e.g., Zinsser et al., 2013, 2016; McLean and Connor, 2015; Sandilos et al., 2015, 2020; Whitaker et al., 2015; Buettner et al., 2016; Roberts et al., 2016; Jeon et al., 2019), and lower job satisfaction (e.g., Carson et al., 2017; Diliberti et al., 2021). For example, in their study of 1001 preschool teachers in 37 Head Start programs in the state of Pennsylvania in the United States, Whitaker et al. (2015) reported that the higher these teachers’ workplace stress, the more conflictual are the teacher-child relationships. Similar, in their study of 1,129 early childhood teachers in center-based childcare programs and public preschools across the United States, Buettner et al. (2016) found that the teachers’ experience of psychological burdens (e.g., depression, stress, emotional exhaustion) predicted their negative reactions to the children’s negative emotions and low levels of professional commitment.

Furthermore, teacher stress has also been correlated with other factors, notably teaching job satisfaction. Teaching job satisfaction, broadly defined as a teacher’s state of feeling content with being a teacher, has been found to correlate negatively with teacher stress in such a way that highly satisfied teachers experience lower levels of stress, whereas teachers who are less satisfied with their teaching condition are more stressed (e.g., Borg et al., 1991; Ho and Au, 2006). The negative correlation between emotional stress and job satisfaction has also been discovered among early childhood teachers in the United States. For example, in their study of 50 United States childcare teachers working with children from low-to-middle class backgrounds, Carson et al. (2017) found that emotional exhaustion was negatively correlated with overall job satisfaction among these teachers.

## Teacher Stress and Stress Coping Mechanisms

Despite the various antecedents of teacher stress, many teachers are still able to become resilient to stress (Howard and Johnson, 2004). There are various models that explain stress resilience. A prominent one is the protective factor model of resilience, suggesting that protective factors (e.g., individual strengths, social support) can counteract the effect of exposure to adversity (Carver, 1998; Prilleltensky et al., 2016). Among these protective resources, social support has been found to be a key contributor to stress resilience (e.g., Gu and Day, 2007; Smith et al., 2008; Day and Gu, 2014; Day and Hong, 2016; Whitebook et al., 2018; Tarrant and Nagasawa, 2020). For instance, Bobek (2002) found that supportive social networks, such as positive school-based relationships with others (e.g., more seasoned teachers, teacher mentors, school leaders, parents), can serve as valuable resources for fostering stress resilience which can, in turn, enhance other aspects of one’s professional life including teaching effectiveness and job satisfaction. Similarly, based on their survey-based study of 3,355 early childhood educators in all types of early childhood settings throughout New York State in the United States during COVID-19, Tarrant and Nagasawa (2020) reported that these educators relied on various mechanisms to help combat stress, with the most prominent one being emotional support from others. Furthermore, Smith et al. (2008) reported that stress resilience was positively correlated with optimism and social support, but negatively associated with perceived stress. Another protective buffer safeguarding individuals’ mental health is gratitude, defined generally as an affective disposition of feeling thankful (McCullough et al., 2002). Gratitude has been found to be associated positively with psychological wellbeing and negatively with burnout among teachers (e.g., Chan, 2010, 2011; Howells and Cumming, 2012).

## Measures of Teacher Stress

Currently, there exist a few occupation-specific measures of teacher stress, such as the “Teacher Stress Inventory” (Fimian, 1986; modified by Fimian and Fastenau, 1990), and the “Index of Teaching Stress” (Greene et al., 1997). However, these measures tend to focus extensively on the sources and manifestations of teacher stress vis-à-vis the school ecology (e.g., quality of teacher-student interactions, time management, student discipline and motivation). There is even a clinical measure (“Index of Teaching Stress,” the same name as Green et al.’s) assessing the stress of teachers teaching students in preschool-12th grade across various teaching stressors in relationship to certain student characteristics (Abidin et al., 2004). Building on and extending these extant teacher stress assessments, we created the new Teacher Stress Scale with items that measure specifically two categories of risk factors as contributing to the teacher feeling “stressed”: (1) lacking support from students’ families, school administrators, colleagues, and own family and friends; and (2) teacher-related demanding circumstances (e.g., too much work, too little time). As teachers are purportedly unique in their stress coping, some may emerge from stress stronger than ever, while some others may merely survive the stress, become resilient from it, or falter in a downward spiral. The question is, how do some teachers manage to thrive despite stress? The answer may lie in the protective resources manifested in these teachers’ capacity to foster thriving dispositions.

## Measures of Psychological Thriving

Empirical support for the validity of thriving has come primarily from health studies especially those concerning chronically ill patients (e.g., Abraído-Lanza et al., 1998; Sirois and Hirsch, 2013; Sirois et al., 2017). Specifically, to our knowledge, Sirois and Hirsch (2013)’s Psychological Thriving Scale is the only one that was formulated to reflect O’Leary and Ickovics’s (1995) and Carver’s (1998) conceptualizations of thriving. However, Sirois and Hirsch’s Psychological Thriving Scale targeted specifically arthritis patients’ perceptions of change (positive or negative), asking them to compare their various current situations to those prior to having arthritis by rating each one on a four-point Likert-type scale according to four interpretative changes: (1) positive change (thriving), (2) no change (resilience), (3) slight negative change, and (4) large negative change. Consequently, despite its empirical contribution, Sirois and Hirsch’s Psychological Thriving Scale focusing on a specific type of adversity (i.e., chronic illness) was not suitable for assessing the teachers’ capacity to thrive in the context of teacher stress.

Furthermore, although research has highlighted thriving as a key marker of psychological wellbeing, teacher thriving appears to be a relatively young field. It may be because reflecting their conceptual affinity, both teacher resilience and thriving are often treated synonymously and even to the extent that teacher thriving is included as part of the resilience discourse or vice versa (e.g., Smith et al., 2008; Beltman et al., 2011; Collie and Perry, 2019). Furthermore, previous studies that did examine thriving specifically in the context of teacher stress (e.g., Sumsion, 2003, 2004; Howard and Johnson, 2004; Malloy and Allen, 2007) have shown little clear distinction between resilience and thriving as they tended to lump the two together. Thus, our understanding of psychological thriving among teachers is inadequate, hampered by a lack of empirical research on teacher thriving. To address this research gap, we developed our own teacher-specific scale, namely Teacher Thriving Scale, to investigate potential thriving dispositions especially among early childhood teachers.

## Teacher Thriving Despite Stress as a Theoretical Framework

O’Leary and Ickovics (1995) proposed four general responses to stress or adversity: (1) succumbing to it, (2) surviving it, (3) recovering or becoming resilient from it, and (4) thriving from it. Building on O’Leary and Ickovics’ theorization, Carver (1998) further elaborated on these four general patterns as representing: (1) a total loss (succumbing to the initial downward turn with continuous deterioration physically and/or psychologically), (2) some loss (surviving it with residual negative impacts), (3) resilience (resuming homeostasis or “bouncing back” to the pre-adversity state), and (4) thriving (surpassing beyond homeostasis with transformative growth). Of all four responses, resilience and thriving are considered optimal because they are both constructive coping mechanisms. However, thriving represents the best scenario because its positive effects go beyond mere resilience. Specifically, Carver (1998) delineated psychological thriving as a positive response to adversity by representing “a kind of growth: growth in knowledge, growth in skill, growth in confidence…” (p. 252). The thriving theory (O’Leary and Ickovics, 1995; Carver, 1998) has contributed a new perspective regarding the treatment of adversity not necessarily as a debilitating, pathological problem, but as an impetus for coping and psychological growth. As we were keen to understand early childhood teachers’ ability to thrive despite stress, the thriving theory served as an appropriate conceptual framework for this study.

## The Goals of This Study

As COVID-19 lingers, research is needed to understand the extent of stress and associated coping manifestations experienced by early childhood teachers who are responsible for educating young children. To address this research need, we developed two new scales, namely the TSS and the TTS. The goal of this study was to conduct a psychometric analysis of these two scales as an essential aspect of the scale development process. Specifically, we focused on (1) evaluating the psychometric properties (validity and reliability) of both the TSS and the TTS, and (2) assessing the internal structures of these two scales. To achieve this goal, we administered our two newly developed scales online to a national sample of early childhood teachers teaching in early grades (preschool through third) in the United States during COVID-19. To this end, the current investigation was guided by the following two overarching research questions in relation to the TSS and the TTS:

1. *How valid is each of the two scales based on its test content, internal structure, and relationship with others measures?*
2. *How reliable is each of the two scales based on its internal consistency and test–retest reliability?*

If both the TSS and the TTS are proven psychometrically sound, it will provide a new empirical framework for viewing teacher stress through a positive coping lens. Furthermore, the demonstration of either the unifactorial or multifactorial structures of the TSS and the TTS will help clarify the conceptual nature of teacher stress and teacher thriving, respectively. Additionally, the nature of the relationships of teacher thriving with other constructs can yield important implications for incorporating cognate factors into the teacher thriving framework when addressing teacher stress. Furthermore, by identifying protective factors influencing teachers’ capacity to thrive, this study can yield insights for incorporating these elements in professional development to promote teacher thriving.

## Materials and Methods

### Scale Development

To contribute knowledge of teacher stress and teacher thriving to the psychology and education fields, we created the TSS and the TTS by undertaking the scale development process. This process included the following five steps (Furr, 2011; Dimitrov, 2012): (1) defining the construct measured, (2) creating a pool of potential scale items using a Likert scale as a response format, (3) seeking feedback on the items from experts, (4) revising, adding, or deleting items as necessary, and (5) administering the finalized scale and evaluating its psychometric properties.

First, the construct measured by the TSS was teacher stress, which was defined by Kyriacou’s (2001) (as highlighted earlier) as a negative emotional experience by a teacher resulting from some aspect of their teaching work. The construct assessed by the TTS was teacher thriving. We followed Carver’s (1998) definition of thriving (as described earlier) as a positive response to adversity by representing “a kind of growth: growth in knowledge, growth in skill, growth in confidence…” (p. 252) but in the context of teaching in our study.

Second, we created a pool of initially seven potential items for the TSS and 25 potential items for the TTS. The TSS was created to assess the extent to which early childhood teachers “felt stressed” regarding a specified aspect of their teaching situation. The development of the items for the TSS was informed by theories (e.g., Lazarus and Folkman, 1984; Kyriacou, 2001) and the extant literature on early childhood teacher stress and stress coping (e.g., Whitaker et al., 2015; Roberts et al., 2016; Clayback and Williford, 2021; Tebben et al., 2021; Chen, 2022; Chen, under review). For instance, the theoretical and empirical body of literature suggests that the lack of social support and high levels of teaching demands are two key sources of teacher stress. Accordingly, we developed items targeting these two areas. Examples of these items are described later. The response format for the scale items involved a five-point Likert scale (1 = Strongly Disagree, 2 = Disagree, 3 = Neutral, 4 = Agree, and 5 = Strongly Agree).

The TTS was developed to assess early childhood teachers’ capacity to thrive despite stress. The development of the items for the TTS was guided by both the thriving theory (e.g., O’Leary and Ickovics, 1995; Carver, 1998) and empirical evidence concerning teacher resilience and thriving (e.g., Sumsion, 2003, 2004; Beltman et al., 2011; Mansfield et al., 2012; Daniilidou and Platsidou, 2018). For example, we developed items for the TTS, reflecting Carver’s (1998) three indicators of thriving: (1) growth in skills and knowledge, (2) growth in confidence, and (3) strengthening of personal relationships. Furthermore, according to Carver, factors contributing to thriving could be clustered around three groups: (1) personal variables, (2) contextual variables, and (3) the interplay between personal and contextual variables. Personal factors may include hope (Snyder, 1994) and optimism (Scheier and Carver, 1992), while contextual factors may involve social support (Gu and Day, 2007; Smith et al., 2008; Castro et al., 2010). Accordingly, some of the items for the TTS also focused on these personal and contextual factors. Examples of the items for the TTS are described later. Just like the TSS, the response format for the TTS items involved a five-point Likert scale (1 = Strongly Disagree, 2 = Disagree, 3 = Neutral, 4 = Agree, and 5 = Strongly Agree).

Third, we subsequently piloted the items for both the TSS and the TTS to three groups of 35 early childhood teachers and consulted two early childhood teacher educators on the clarity and representativeness of the construct content. Fourth, incorporating feedback on issues from face validity to item wording from these two groups of early childhood educators, we made some relevant revisions to the original scales including rephrasing ambiguous items, deleting some items with duplicate ideas, and adding more relevant items. This process led to our finalization of the TSS as comprising nine items and the TTS as involving 20 items.

The TSS’s nine items represented nine situational stressors triggering the teachers to feel stressed. The items included, “I felt stressed for not having support from the administrators at my school.” and “I felt stressed for not having enough time to complete my teaching work (e.g., preparing, teaching the curricular content)” (see Table 1 for the entire list of the items). As shown in Table 1, our original hypothesis was that all nine items of the TSS belonged to two latent factors (F1 = Lacking Social Support, and F2 = Teaching-related Demands).

**TABLE 1:** The Teacher Stress Scale’s (TSS) original 2-factor (F) design and response category distribution.

	Item ID		Strongly disagree	Disagree	Neutral	Agree	Strongly agree
F1	TSS1	I felt stressed when encountering issues with my students’ families.	1.60%	27.00%	19.70%	41.00%	3.30%
F1	TSS2	I felt stressed for not having support from the administrators at my school.	12.30%	21.30%	18.90%	19.70%	20.50%
F1	TSS3	I felt stressed for not having support from colleagues at my school.	16.40%	32.00%	11.50%	25.40%	7.40%
F1	TSS4	I felt stressed for not having support from my family and friends.	19.70%	45.10%	14.80%	10.70%	1.60%
F1	TSS5	I felt stressed for having to manage student behaviors.	5.70%	28.70%	23.00%	21.30%	12.30%
F2	TSS6	I felt stressed for having too much teaching work to do.	3.30%	19.70%	18.00%	27.90%	23.00%
F2	TSS7	I felt stressed for not having enough time to complete my teaching work (e.g., preparing, teaching the curricular content).	3.30%	19.70%	12.30%	30.30%	26.20%
F2	TSS8	I felt stressed for not being able to meet the diverse learning needs of my students.	4.90%	28.70%	16.40%	26.20%	16.40%
F2	TSS9	I felt stressed about not doing a good job with my teaching.	9.00%	32.80%	13.90%	27.00%	9.00%

The TTS’s 20 items represented 20 personal and contextual attributes including adaptability (e.g., “I was able to successfully adapt to the stress of my teaching situation.”), professional growth (e.g., “I treated challenges of my teaching situation as opportunities for professional learning and growth.”), and positive mindset (e.g., “I was optimistic about continuing my job as a teacher.”) (see Table 2 for the full list of the items). As shown in Table 2, our original assumption was that the 20 items comprising the TTS belonged to six latent factors (F1 = Adaptability and Flexibility, F2 = Creativity and Resourcefulness, F3 = Locus of Control and Coping Efficacy, F4 = Professional Learning and Growth, F5 = Confidence, Hope, Patience, and Perseverance, and F6 = Job Satisfaction).

**TABLE 2:** The Teacher Thriving Scale’s (TTS) original 6-factor (F) design and response category distribution.

	Item ID		Strongly disagree	Disagree	Neutral	Agree	Strongly agree
F1	TTS1	I was able to successfully adapt to the stress of my teaching situation.	3.30%	16.40%	18.90%	52.50%	8.20%
F1	TTS2	I was able to successfully adapt to any instructional modality.	0.80%	6.60%	18.90%	61.50%	11.50%
F1	TTS3	I was able to be flexible with my teaching method and strategies to accommodate stressful changes in my teaching situation.	0.80%	8.20%	8.20%	62.30%	19.70%
F2	TTS4	I was able to be creative with my teaching method and strategies with whatever resources I had.	0.80%	4.10%	10.70%	60.70%	23.00%
F2	TTS5	I was able to integrate technology in my lessons in innovative ways.	1.60%	7.40%	20.50%	51.60%	17.20%
F2	TTS6	I sought resources and professional development opportunities to enhance my ability to successfully cope with the stress of my teaching situation.	1.60%	10.70%	20.50%	44.30%	22.10%
F2	TTS7	I sought resources and opportunities to learn new pedagogical knowledge and skills to apply to better cope with stressful teaching situations.	1.60%	14.80%	21.30%	44.30%	17.20%
F3	TTS8	I did not dwell on the stress of my teaching situation (that I had no control over).	4.90%	32.80%	22.10%	34.40%	4.10%
F3	TTS9	I was able to successfully cope with the stress of my teaching situation (that I had control over).	3.30%	10.70%	23.00%	55.70%	6.60%
F3	TTS10	I believe in my ability to successfully cope with the stress of my teaching situation (that is within my control) in the future.	0.80%	7.40%	26.20%	52.50%	11.50%
F4	TTS11	I treated challenges of my teaching situation as opportunities for professional learning and growth.	0.80%	4.10%	14.80%	55.70%	19.70%
F4	TTS12	I gained new knowledge, skills, and/or confidence each time that I overcame a stressful teaching situation.	0.00%	3.30%	7.40%	53.30%	30.30%
F4	TTS13	I became a stronger person each time that I overcame a stressful teaching situation.	0.80%	4.10%	15.60%	51.60%	23.00%
F4	TTS14	I applied what I learned from overcoming the stress of my teaching situation to new teaching circumstances.	0.80%	4.10%	17.20%	59.00%	13.90%
F5	TTS15	Overcoming the stress of my teaching situation made me feel more confident in my ability to successfully cope with the stress of my teaching situation in the future.	1.60%	10.70%	17.20%	47.50%	17.20%
F5	TTS16	Support from others through stressful teaching situations made me feel more confident about counting on them for support in the future.	3.30%	8.20%	17.20%	43.40%	23.00%
F5	TTS17	No matter how stressful the teaching situation, I was hopeful that it would get better if I persevered with patience.	1.60%	9.80%	14.80%	50.00%	18.90%
F5	TTS18	I did not give up teaching when it got stressful.	1.60%	1.60%	5.70%	44.30%	41.80%
F6	TTS19	I was happy with my job as a teacher even when I was faced with the stress of my teaching situation.	4.10%	5.70%	21.30%	36.90%	27.00%
F6	TTS20	I was optimistic about continuing my job as a teacher.	4.90%	10.70%	17.20%	32.80%	29.50%

During the fifth and last step of the scale development process, we administered the two newly created scales to participants as described in the “Data Collection” section and then evaluated their psychometric properties systematically as elucidated in the “Data Analysis” section.

### Data Collection

To evaluate the psychometric prosperities of the TSS and the TTS, we first collected data for the two scales through a questionnaire. This questionnaire was administered online to early childhood teachers using Qualtrics (a data collection service^1^) in July 2021. The questionnaire consisted of two sections. The first section included 17 items, asking about the teacher’s sociodemographic background (e.g., age, gender, educational level, years of teaching experience, grade taught, sociodemographic characteristics of the school and their students). The second section comprised 60 items across eight scales, including the two newly developed scales and six preexisting scales in the literature as described in the “Measuring Convergent and Discriminant Evidence for Construct Validity” section. This questionnaire took participants 10–20 min to complete. To encourage participation, participants were invited to enter a lottery to win one of two $50 Amazon e-gift cards.

For the purpose of evaluating the test–retest reliability of the TSS and the TTS, only the TSS and the TTS along with the sociodemographic section were re-administered to willing participants from Time 1 of data collection. Although all participants were invited to participate in this focused follow-up questionnaire, only some of them accepted our invitation to do so. This Time 2 of data collection followed the same administration procedure as that of Time 1 of data collection and, the period between these two times of data collection was about a month. The follow-up questionnaire took participants approximately 5 min to complete. As an incentive for participation, participants were invited to enter a lottery to win one of two $25 Amazon gift cards.

To ensure contextual consistency across all of the scales for both the questionnaires (the original and the follow-up), we asked the participants to respond to each statement in relation to the immediate past 2020–2021 school year. We also checked and double checked the content, format, and design of each questionnaire as well as completed it a few times ourselves as mock trials to ensure accuracy, readability, and accessibility. Any emerging issues were addressed immediately by the research team.

A direct link to each of the two online questionnaires was provided to potential participants. Upon clicking into the questionnaire environment, these potential participants would be immediately directed to the landing page showing an informed consent letter that described the research study including participant involvement and rights. If the individuals agreed to participate, they would then click the statement: “I have read this informed consent letter and understand its contents. By checking this box, I am indicating consent to participate in this research as described.”

### Participant Recruitment

The participants were recruited primarily via HELLO, which is an online platform for members of the National Association for the Education of Young Children (NAEYC) (the largest national professional association for early childhood educators in the United States) to connect, dialogue, and exchange ideas with one another around issues concerning early learning and education^2^. Upon approving our request for assistance with participant recruitment, NAEYC helped post the information about our study along with an invitation link to the original questionnaire on its HELLO platform twice, initially in mid-July and then as a reminder at the end of July 2021. Additionally, the research team members recruited teacher acquaintances by sharing the same information. The only inclusion criterion was that the participants must have taught in an early childhood grade (preschool-third) during the immediate past 2020–2021 school year in the United States amidst COVID-19, given that this contextual information was the focus of our investigation. Our data collection was strategically planned to occur in the summer (July and August) when the teachers would have most likely had time to reflect on their teaching experience during the immediate past school year and relatedly when their memory of that experience would have still been fresh to recall.

### Participants (Entire Sample)

Table 3 summarizes the sociodemographic characteristics of the entire sample from Time 1 (the first wave of data collection in July) and its subsample in Time 2 (the second wave of data collection mostly in August). During Time 1, the participants consisted of a national sample of 122 early childhood teachers teaching preschool to third grade in 26 states of the United States during the 2020–2021 school year. About half of the participants (*n* = 65) were from New Jersey which is not surprising, considering that this state was the home of the research team who would have had easier access to recruiting participants via their professional connections. Despite our efforts to recruit participants, they only resulted in a relatively small sample size of 122 early childhood teachers. This outcome might have partly reflected COVID-19-related challenges and stress confronting other teachers in finding the time and mental space to participate in this study about the very issue with which they might have been struggling: teacher stress.

**TABLE 3:** The sociodemographic characteristics of participants for both Time 1 (*N* = 122) and Time 2 (*N* = 48).

		Time 1		Time 2	
		*N*	%	*N*	%
Gender	Female	111	91.00%	46	95.80%
	Male	8	6.60%	1	2.10%
	Other	2	1.60%	0	0.00%
	Missing	1	0.80%	1	2.10%
Race	White	78	63.90%	27	56.30%
	Hispanic	25	20.50%	12	25.00%
	Asian	4	3.30%	2	4.20%
	African American/Black	9	7.40%	6	12.50%
	Other	3	2.50%	1	2.10%
	Missing	3	2.50%	0	0.00%
Education	Bachelor’s degree	63	51.60%	25	52.10%
	Master’s degree	46	37.70%	19	39.60%
	Doctoral degree	3	2.50%	1	2.10%
	Other	9	7.40%	3	6.30%
	Missing	1	0.80%	0	0.00%
Certification	Early childhood (P-3)	58	47.50%	25	52.10%
	Elementary	23	18.90%	6	12.50%
	Not certified yet	17	13.90%	7	14.60%
	Other	22	18.00%	10	20.80%
	Missing	2	1.60%	0	0.00%
Grade level taught	Preschool	48	39.30%	19	39.60%
	Prekindergarten	24	19.70%	13	27.10%
	Kindergarten	10	8.20%	5	10.40%
	First grade	6	4.90%	4	8.30%
	Second grade	9	7.40%	1	2.10%
	Third grade	9	7.40%	2	4.20%
	Other	15	12.30%	3	6.30%
	Missing	1	0.80%	1	2.10%
School community	Urban	55	45.08%	24	50.00%
	Suburban	63	51.63%	20	41.67%
	Rural	3	2.50%	4	8.33%
	Missing	1	0.82%	0	0.00%
Students’ income background	Low-income	52	42.62%	22	45.83%
	Middle-income	52	42.62%	21	43.75%
	High-income	16	13.12%	5	10.42%
	Missing	2	1.64%	0	0.00%

The entire sample of 122 early childhood teachers aged between 22 and 72 years (*M* = 41). Their teaching experience ranged from 1 to 40 years (*M* = 13). The teachers were teaching primarily in preschool (39.30%) followed by prekindergarten (19.70%). Most of these teachers were female (91.00%) and were White (63.90%). These gender and racial particulars of early childhood teachers align with the general national trend in the United States in 2020 (e.g., 98.8% and 82.8% of preschool and kindergarten teachers were female and White, respectively) (U.S. Bureau of Labor Statistics, 2021).

Education-wise, most of the participating teachers were Bachelor’s degree holders (51.60%) followed by Master’s degree holders (37.70%). Nearly half of the teachers held a state-awarded teaching certificate in Early Childhood (preschool-third grade) (47.50%), 18.90% were certified in Elementary Education, and 13.90% had not yet attained teaching certification. The teachers were concentrated mostly in suburban (51.63%) and urban (45.08%) communities and teaching mostly children from either low-income (42.62%) or middle-income backgrounds (42.62%).

### Participants (Subsample)

Of the 122 original participants, 72 were willing to be contacted via email for participation in the follow-up online questionnaire at Time 2. Although all 72 early childhood teachers were sent an invitation for participation, only 48 completed the follow-up questionnaire. Table 3 summaries the descriptive characteristics of this subsample of teachers. These teachers came from 15 states of the United States, about half of them from New Jersey. They aged between 23 and 66 years (*M* = 40). Their teaching experience ranged from 1 to 40 years (*M* = 11). The teachers were teaching primarily in preschool (39.60%) followed by prekindergarten (27.10%). The majority of these teachers were female (95.80%) and were White (56.30%).

### Measuring Convergent and Discriminant Evidence for Construct Validity

Our aforementioned literature review indicates that teacher thriving is related positively to cognate concepts, especially resilience, resilience coping, coping efficacy, emotional support, teaching job satisfaction, and gratitude, and that teacher stress and teacher thriving are conceptually opposite. Thus, we hypothesized that teacher thriving and its cognate concepts would be correlated positively and that teacher thriving and teacher stress would be correlated negatively. If so, the positive correlations between the teacher thriving and other related concepts would provide convergent evidence for construct validity and the negative correlation between teacher thriving and teacher stress would provide discriminant evidence for construct validity. Accordingly, in addition to the TSS and the TTS, we employed the following six conceptually relevant and previously validated scales:

1. *Resilience.* Smith et al.’s (2008) Brief Resilience Scale (BRS) consists of six items, with three being positive (e.g., “I tend to bounce back quickly after hard times.”) and three being negative that were reverse coded (e.g., “It is hard for me to snap back when something bad happens.”).
2. *Resilience Coping.* Sinclair and Wallston’s (2004) Brief Resilient Coping Scale (BRCS) comprises four items (e.g., “I look for creative ways to alter difficult situations.” “I believe I can grow in positive ways by dealing with difficult situations.”).
3. *Coping Efficacy.* Three items are adapted from Gignac et al.’s (2000) Coping Efficacy Scale (CES) to reflect the teaching situation (i.e., “I successfully coped with the stress of my teaching condition.” “I successfully coped with the day-to-day teaching-related challenges.” and “I successfully coped with the emotional aspects of teaching-related stress.”).
4. *Emotional Support.* Shakespeare-Finch and Obst’s (2011) Receiving Emotional Support Scale (RESS), a subscale of their Social Support Scale, comprises seven items (e.g., “There is someone I can talk to about the pressures in my life.” “There is at least one person that I can share most things with.”).
5. *Teaching Satisfaction.* Ho and Au’s (2006) Teaching Satisfaction Scale (TESS) includes five items (e.g., “My conditions of being a teacher are excellent.” “I am satisfied with being a teacher.”).
6. *Gratitude.* McCullough et al.’s (2002) Gratitude Questionnaire-6 (GQ-6) consists of six items, with four being positive (e.g., “I have so much in life to be thankful for.” “I am grateful to a wide variety of people.”), and two being negative that are reversed coded (i.e., “When I look at the world, I don’t see much to be grateful for.” “Long amounts of time can go by before I feel grateful to something or someone.”).

## Data Analysis

### Scale Validity Testing

We investigated three types of validity for the TSS and the TTS: (1) content validity, (2) internal structure, and (3) construct validity. First, in psychometric testing, content validity refers to the extent to which a measure represents relevant aspects of the targeted construct (Haynes et al., 1995). The aspects of content validity that we focused on included the clarity of wordings, representativeness of the items in assessing the content of the construct, and the adequacy of the scale format (Haynes et al., 1995; Koller et al., 2017).

Second, according to the measurement standards recommended by American Educational Research Association [AERA] (2014), it is essential that we examine the internal structure of a scale as evidence of validity because it shows whether the scale’s total score, domain scores, or both convey trustworthy estimations of latent traits. To assess the internal structure of the TSS and of the TTS, we ran a multi-step analysis. In Step 1, we examined the original design of the scales through confirmatory factor analysis (CFA): a 2-factor model for TSS and a 6-factor model for TTS (see Tables 1, 2, respectively). In Step 2, if the original design did not meet the model fit criteria, we would proceed to conducting exploratory factor analysis (EFA) to seek a better scale structure as determined by the model fit. We planned to eliminate any items or latent factors that would not meet the general criteria for scale development. These criteria are described in detail in the following paragraphs. In Step 3, we used CFA to confirm and finalize the scale structure identified in Step 2. All internal structure analyses were completed in Mplus 8.3 (Muthén and Muthén, 1998–2021). The weighted least square mean and variance adjusted (WLSMV) estimator was used to extract factors because they accounted for the ordinal and polytomous nature of the item responses for both the TSS and the TTS.

To evaluate the model fit, we adopted the following criteria: (1) the chi-square statistic (a *p*-value of 0.005 or larger is considered acceptable but it is not always an accurate determinator because of other factors) (e.g., sample size, number of variables) (Jöreskog, 1969; Muthén and Muthén, 1998–2021; Wang and Wang, 2012); (2) comparative fit index [CFI; 95 or higher for good model fit, and 0.90 or greater is considered acceptable (Bentler, 1990)], and the Tucker–Lewis index [TLI; 0.95 or higher for good model fit, and 0.90–0.94 for acceptable model fit (Tucker and Lewis, 1973)]; as well as (3) the root-mean-square error of approximation [RMSEA; a value of 0.05 or less suggests good fit, values between 0.05 and 0.08 acceptable fit, values between 0.08 and 0.10 mediocre fit, and values greater than 0.10 poor fit (Browne and Cudeck, 1992)], and the standardized root-mean-square residual [SRMR; a value of 0.08 or lower for good model fit (Browne and Cudeck, 1992; Brown, 2006; Asparouhov and Muthén, 2018)].

From the perspective of internal validity, to assess the quality of items and domains for both the TSS and the TTS, we adopted the following rules from Hair et al. (2019): (1) our study met the preferred minimum sample size for factor analysis, which should be 100 or larger; (2) the scales met the desired ratio of at least five observations per item (i.e., nine items for the TSS and 20 items for the TTS); (3) at the EFA step, the minimally acceptable factor loadings should be 0.4 for small sample sizes; (4) to handle cross-loading items detected from EFA, we further examined and compared the improvement of model fit between when an item was considered in one factor or when it was deleted. In the finalized scale model structure, we expected that (1) each factor should have at least three items with high loadings; and (2) to define high factor loadings for our sample size of 122, we considered those that exceeded 0.50 (Hair et al., 2019).

The third category of validity that we examined was construct validity. Specifically, we tested two subtypes of evidence: (1) convergent evidence, and (2) discriminant evidence. We did both by comparing the scales with other previously validated instruments that were conceptually cognate or opposite. We adopted the multi-trait multi-method (MTMM) design (Campbell and Fiske, 1959) to establish evidence for convergent validity. Convergent evidence refers to the relationships between the scores of a targeted assessment and those of other assessments purported to measure the same or similar constructs (American Educational Research Association [AERA], 2014). In this study, the TTS was compared with six conceptually relevant and methodologically validated scales (BRS, BRCS, CES, RESS, TESS, and GQ-6). The degree of their convergence would be indicated by Pearson’s correlation coefficients. If the TTS correlated significantly in the positive direction with the other scales as hypothesized, we would conclude that the TTS had adequate convergent evidence. Furthermore, given that the TSS and the TTS were conceptually opposite, we hypothesized that they would be negatively related to each other. Such a result would provide discriminant evidence for construct validity of these two scales.

### Scale Reliability Testing

To establish scale reliability, we focused on two types: (1) internal consistency, and (2) test–retest reliability. According to the standards set by American Educational Research Association [AERA] (2014), for each test’s total score or domain scores that are being used, estimates of reliability should be reported. Thus, in this step, we tested the degree of internal consistency for the TSS and the TTS. Internal consistency is defined as an assessment of “the extent to which all the items in a test measure the same concept or construct and hence it is connected to the inter-relatedness of the items within the test” (Tavakol and Dennick, 2011, p. 53). Applying the traditional reliability measure using Cronbach’s alpha (Cronbach, 1951), we calculated the internal consistency of the items in each subscale and the overall scale of the TSS and of the TTS. The Cronbach’s alpha coefficients range from 0 to 1, with the higher the value, the stronger the inter-correlation (Tavakol and Dennick, 2011), and with acceptable internal reliability requiring a minimum value of 0.70 (Nunnally, 1978; Nunnally and Bernstein, 1994).

The test–retest reliability of the TSS and the TTS was estimated from the subsample of teachers who participated in both Time 1 and Time 2 of data collection. To evaluate test–retest reliability, we inspected the correlations between the two sets of results for the TSS and those for the TTS. A Pearson correlation coefficient of a minimum of 0.70 is considered acceptable test–retest reliability to indicate a general relationship strength (Nunnally and Bernstein, 1994).

## Results

### Content Validity

A two-step process helped establish the content validity of the TSS and the TTS. First, as described earlier, we formulated the items on the basis of theoretical and empirical evidence. Second, as also detailed earlier, we then piloted the items to 35 early childhood teachers and consulted two early childhood teacher educators on the extent to which these items measured what they were intended and if they were phrased unambiguously. Upon receiving feedback from these individuals, we revised the scale items accordingly including rephrasing some wordings, adding items, and deleting duplicate words or items to achieve better content validity.

### Internal Structure Validation of the Scales

We first reviewed the distribution of all item response categories (“Strongly Disagree,” “Disagree,” “Neutral,” “Agree,” and “Strongly Agree”) for both the TSS and the TTS. All five categories of the nine items for the TSS were reasonably used. Thus, we kept the original response categories for TSS for the following analyses (see Table 1). However, for the TTS scale, the percentage of responses with “Strongly Disagree” was below 5% for all 20 items, and most of the cases only had around 1% reporting “Strongly Disagree.” Given this indication, we decided to combine the “Strongly Disagree” and “Disagree” categories to maintain the meaningfulness of the response categories for a later analysis (see Table 2).

In Step 1 of the internal structure validation, we tested the 2-factor model for the TSS and the 6-factor model for the TTS as originally designed. Tables 1, 2 summarize the original item-factor structures for the TSS and the TTS separately. Table 4 shows the goodness-of-fit indices of the 2-factor CFA model for the TSS: (1) chi-square = 80.73 (df = 26, *p* < 0.00), (2) CFI = 0.97, (3) TLI = 0.96, (4) RMSEA = 0.14, and (5) SRMR = 0.06. These results showed an acceptable to good level of model fit across all indices. As the original design was confirmed, we skipped Step 2 for the TSS. However, we did notice that two items (TSS1 and TSS4) had factor loadings below 0.5 (see Table 5 for detailed factor loadings of the 2-factor CFA model). Therefore, we could not finalize the scale with two items that did not have high enough factor loadings. Upon content review, we noticed that TSS1 and TSS4 were sourcing stress from students or teachers’ families, while other items were more directly related to school- or classroom-based stress. It may be that TSS1 and TSS4 were not testing the similar latent content as TSS2, TTS3, and TTS5. Considering the CFA results, the 0.5 factor loading rule, and content review, we decided that it was better to delete Items TSS1 and TSS4 for modification.

**TABLE 4:** Model fit indices for the TSS internal structure validation.

		Number of parameters	Chi-square	df	*p*	RMSEA	CFI	TLI	SRMR
Step 1: CFA	Original 2-factor CFA model	46	80.73	26	<0.001	0.14	0.97	0.96	0.06
Step 3: CFA	New 2-factor CFA model	36	61.68	13	<0.001	0.18	0.98	0.96	0.06

**TABLE 5:** Factor loadings for the TSS as identified by the original 2-factor CFA model.

	F1	F2
TSS1	0.499**	
TSS2	0.85**	
TSS3	0.79**	
TSS4	0.43**	
TSS5	0.61**	
TSS6		0.96**
TSS7		0.94**
TSS8		0.83**
TSS9		0.72**

**Significant at the 1% level.

Additionally, Table 6 presents the goodness-of-fit indices of the 6-factor CFA model for the TTS according to our original design: (1) chi-square = 374.48 (df = 160, *p* < 0.00), (2) CFI = 0.89, (3) TLI = 0.87, (4) RMSEA = 0.11, and (5) SRMR = 0.90. This model showed poor fit, suggesting that the original design needed to be modified for the TTS. Consequently, we proceeded to Step 2.

**TABLE 6:** Model fit indices for the TTS internal structure validation.

		Number of parameters	Chi-square	df	*p*	RMSEA	CFI	TLI	SRMR
Step 1: CFA	Original 6-factor model	90	374.48	160	<0.001	0.11	0.89	0.87	0.09
Step 2: EFA	New 6-factor model	105	106.10	85	0.06	0.05	0.99	0.98	0.04
Step 3: CFA	Solution 1	51	109.10	51	<0.001	0.10	0.96	0.95	0.06
	Solution 2	51	128.61	51	<0.001	0.11	0.95	0.94	0.06

In Step 2, we conducted EFA for TTS to explore better model structures. The EFA results for the TTS indicated that the 6-factor model reached a good level of model fit. Table 6 shows the goodness-of-fit indices of the new 6-factor model included: (1) chi-square = 106.10 (df = 85, *p* = 0.06), (2) CFI = 0.99, (3) TLI = 0.98, (4) RMSEA = 0.045, and (5) SRMR = 0.035. As shown in Table 7, we did notice that despite the good model fit, several issues required modification consideration. First, items TTS5 and TTS16 were not well loaded (below 0.40) on any of the latent factors so we decided to eliminate both items. Second, two latent factors, FA4 and FA5 (from the new set of notations to differentiate it from F4 and F5 from the original set of notations), only had two items loaded at the most, which might cause generalizability and stability issues for the entire scale. This result indicated that we might not have an adequate number of items to configure these two latent constructs, leading us to eliminate both factors. Third, as shown in Table 7, the three items (TTS10, TTS11, and TTS17) had questionable cross-loading issues. Specifically, Item TTS10 had a pair of close loadings on FA2 and FA6, Item TTS11 had a pair of close loadings on FA1 and FA2, and Item TTS17 had a pair of close loadings on FA1 and FA3. Our theoretical judgment led us to believe that TTS11 would be best suited with the other items in FA2, and similarly TTS17 suited in FA3. Although allowing cross-loading might create a good fit, it would not meet our needs to confirm generic measurement representation. While Item TTS3 might also have cross-loading issues on FA1 and FA4, since FA4 had already been deleted so we decided to keep it under F1 for further evaluation. We also decided then to use CFA to select a better solution in Step 3.

**TABLE 7:** Factor loadings for the TTS as identified by the new 6-factor EFA model.

	FA1	FA2	FA3	FA4	FA5	FA6
TTS1	0.58*	0.14	0.17	0.00	0.004	0.18*
TTS2	0.79*	–0.08	–0.03	0.18	0.10	0.03
TTS3	0.47*	–0.07	0.004	0.50*	0.17*	0.04
TTS4	–0.05	0.02	0.09	0.88*	–0.02	0.08
TTS5	0.18*	0.35*	0.001	0.37*	0.03	−0.22*
TTS6	0.13	–0.04	0.09	0.07	0.78*	−0.13*
TTS7	–0.10	0.06	–0.03	–0.06	0.89*	0.14
TTS8	–0.04	–0.15	–0.02	0.10	0.16*	0.68*
TTS9	0.04	0.03	0.08*	0.03	0.02	0.99*
TTS10	0.17*	0.36*	–0.001	0.13	–0.03	0.48*
TTS11	0.52*	0.34*	0.13	0.01	–0.05	0.04
TTS12	0.26*	0.55*	0.02	0.12	0.27*	–0.12
TTS13	–0.05	0.75*	0.22*	0.06	0.14*	–0.05
TTS14	0.01	0.65*	–0.05	0.24*	0.06	0.24*
TTS15	0.16	0.58*	0.11	–0.08	–0.02	0.22*
TTS16	0.30*	0.38*	0.13	−0.18*	0.006	0.15
TTS17	0.52*	0.14	0.34*	−0.19*	–0.005	0.20*
TTS18	0.30*	0.21*	0.42*	0.12	–0.05	–0.10
TTS19	0.01	0.13	0.67*	0.11*	–0.04	0.09
TTS20	–0.03	−0.05*	1.06*	0.02	0.05	0.03

*Factor loadings are emboldened if their magnitude is over 0.4. Items below a 0.4 loading are considered for deletion. Items TTS10, TTS11, and TTS17 are subject to further examination for cross-loadings.*

*Significant at the 5% level.

In Step 3, we tested the modified 7-item TSS structure with all items loaded onto its original latent factor design through CFA. Table 4 shows that the goodness-of-fit indices of the new 2-factor model of TSS reached acceptable to good level of model fit: (1) chi-square = 61.68 (df = 13, *p* < 0.00), (2) CFI = 0.98, (3) TLI = 0.96, (4) RMSEA = 0.18, and (5) SRMR = 0.05. All items were loaded high on the detected latent factors (i.e., greater than 0.5). Table 8 shows the items for each of the two constructs. Based on the factor analysis results, the three items (TSS2, TSS3, and TSS5) were assumed to measure a similar latent construct: Inadequate School-based Support. The subscale score (TSS-F1) was calculated as the mean of these three items. We define the construct, Inadequate School-based Support, as the lack of support from individuals at the school level (i.e., administrators, colleagues, students). The four items (TSS6, TSS7, TSS8, and TSS9) were assumed to measure a similar latent construct: Teaching-related Demands. The subscale score (TSS-F2) was calculated as the mean of these four items. We define the construct, Teaching-related Demands, as the nature and amount of teaching work that exceed the teacher’s capacity to manage such work without pressure. The TSS overall scale score (TSS-All) is the mean of all reserved seven items.

**TABLE 8:** Finalized internal structure of the TSS and the TTS through CFA.

		Factor loading	Standard error	*p*
**Teacher Stress Scale**				
**TSS-F1: Inadequate School-based Support BY**				
	TSS2	0.87	0.05	<0.001
	TSS3	0.81	0.06	<0.001
	TSS5	0.63	0.08	<0.001
**TSS-F2: Teaching-related Demands BY**				
	TSS6	0.95	0.02	<0.001
	TSS7	0.94	0.03	<0.001
	TSS8	0.82	0.07	<0.001
	TSS9	0.72	0.05	<0.001
**Teacher Thriving Scale**				
**TTS-F1: Adaptability and Flexibility BY**				
	TTS1	0.86	0.04	<0.001
	TTS2	0.78	0.04	<0.001
	TTS3	0.69	0.06	<0.001
**TTS-F2: Personal strengths and Professional Growth BY**				
	TTS11	0.83	0.05	<0.001
	TTS12	0.81	0.05	<0.001
	TTS13	0.82	0.05	<0.001
	TTS14	0.75	0.06	<0.001
	TTS15	0.75	0.05	<0.001
**TTS-F3: Positive Mindset BY**				
	TTS17	0.84	0.05	<0.001
	TTS18	0.77	0.06	<0.001
	TTS19	0.81	0.04	<0.001
	TTS20	0.84	0.04	<0.001

Additionally, in Step 3, we conducted a series of CFA to assess the modified structure of TTS after deleting the problematic items and factors. We further noticed that items TTS9 and TTS10 encountered a collinearity issue. However, deleting one of them would cause the corresponding latent factor (FA4) (see Table 7) to have only two items. Therefore, after careful consideration, we decided to eliminate the entire FA6 latent factor to maintain the stability and generalizability of the TTS scale. Consequently, we ended up with three factors (FA1, FA2, and FA3 as shown in Table 7) across 12 items based on previous steps.

The last issue we sought to solve was the cross-loading problem of Items TTS11 and TTS17. Two CFA models were tested and compared to help us determine the attributions of Items TTS11 and TTS17. In CFA Solution 1, we took the data-driven approach by setting Items TTS1 to TTS3 along with Items TTS11 and TTS17 to represent FA1, Items TTS12 to TTS15 to represent FA2, and Items TTS18 to TTS20 to group as FA3. As a comparison, in CFA Solution 2, we took the theory- driven approach, resulting in Items TTS1 to TTS3 to represent FA1; Item TTS11 joining Items TTS12 to TTS15 to represent FA2, and Item TTS17 merging with Items TTS18 to TTS20 to group as FA3.

Table 6 presents the model fit indices for Solution 1 and Solution 2 for the TTS internal structure validation. Although Solution 1 exhibited slightly better fit statistically, Solution 2 also reached an acceptable level of model fit according to CFI and made a much better theoretical sense in terms of explanation of the latent factors. For these reasons, we decided to finalize the TTS scale structure with Solution 2.

Table 8 presents the items for each construct, which indicated that the finalized 3-factor TTS model had high factor loadings for all items. Based on the factor analysis results, Items TTS1, TTS2, and TTS3 were assumed to measure a similar latent construct: Adaptability and Flexibility. The subscale score (TTS-F1) was calculated as the mean of these three items. We define the construct, Adaptability and Flexibility, as the teacher’s capacity to adjust to changing teaching situations. The five items (TTS11, TTS12, TTS13, TTS14, and TTS15) were assumed to measure a similar latent construct: Personal Strengths and Professional Growth. The subscale score (TTS-F2) was calculated as the mean of these five items. We define the construct, Personal Strengths and Professional Growth, as the personal attributes and professional growth in knowledge, skills, and confidence that enable the teachers to thrive in their teaching work. Items TTS17, TT18, TTS19, and TTS20 were assumed to measure a similar latent construct: Positive Mindset. The subscale score (TTS-F3) was calculated as the mean of the aforementioned five items. The construct, Positive Mindset, is defined as the teachers’ ability to exhibit a positive outlook on their teaching work and themselves as teachers supported by their personal attributes including hope, perseverance, and optimism. Finally, the TSS overall scale score (TTS-All) was calculated as the mean of all reserved 12 items.

### Convergent and Discriminant Evidence for Construct Validity

Confirming our hypothesis about the potential convergence of the TTS and other cognate scales, Table 9 shows that the overall TTS was correlated positively and significantly with all of the six convergent scales: (1) with BRS (*r* = 0.44, *p* < 0.01), (2) with BRCS (*r* = 0.48, *p* < 0.01), (3) with RESS (*r* = 0.42, *p* < 0.01), (4) with CES (*r* = 0.69, *p* < 0.01), (5) with TESS (*r* = 0.54, *p* < 0.01), and (6) with GQ-6 (*r* = 0.38, *p* < 0.01). Additionally, Table 5 displays the correlations of the subscales with the convergent scales, demonstrating that all TTS subscales were also significantly and positively correlated with each of the six convergent scales, albeit some pair correlations were weak (e.g., TTS-F1 with RESS and GQ-6). However, while the overall TSS was not correlated with BRCS, RESS, or GQ-6, it was negatively correlated with BRS, CES, and TESS. These results provide discriminant evidence for construct validity for the TSS, which is not surprising because we expected the TSS to test different latent structure from the six scales.

**TABLE 9:** Pearson correlation coefficients for the TTS convergent validity.

	BRS	BRCS	RESS	CES	TESS	GQ-6
TSS-F1	−0.25**	–0.03	–0.07	–0.17	−0.21*	−0.24*
TSS-F2	–0.14	–0.06	0.06	−0.29**	−0.40**	–0.003
TSS-ALL	−0.23*	–0.05	–0.004	−0.27**	−0.36**	–0.14
TTS-F1	0.31**	0.34**	0.24**	0.50**	0.41**	0.20*
TTS-F2	0.42**	0.49**	0.37**	0.62**	0.38**	0.32**
TTS-F3	0.40**	0.41**	0.45**	0.66**	0.57**	0.44**
TTS-ALL	0.44**	0.46**	0.42**	0.69**	0.54**	0.38**

*Significant at the 5% level.

**Significant at the 1% level.

As hypothesized, the overall TSS and the overall TTS were significantly and negatively correlated (*r* = −0.29, *p* < 0.01), and the subscales of the TSS and those of the TTS were correlated either negatively or not significantly (see Table 10). The negative correlation between the overall TSS and the overall TTS provides discriminant evidence for each other’s construct validity. Furthermore, Table 10 shows that the overall TSS and its subscales were significantly and positively correlated, and similarly the overall TTS and its subscales were also significantly and positively correlated. These inter-correlations provide evidence that the items are assessing the same content (Cohen and Swerdlik, 2013).

**TABLE 10:** Correlations among the TSS and the TTS and their subscales.

	TSS-F1	TSS-F2	TSS-ALL	TTS-F1	TTS-F2	TTS-F3	TTS-ALL
TSS-F1	1.00						
TSS-F2	0.46**	1.00					
TSS-ALL	0.86**	0.85**	1.00				
TTS-F1	−0.29**	−0.23*	−0.31**	1.00			
TTS-F2	−0.20*	–0.09	–0.17	0.61**	1.00		
TTS-F3	−0.29*	–0.13	−0.25**	0.58**	0.65*	1.00	
TTS-ALL	−0.31**	–0.18	−0.29**	0.86**	0.86**	0.88**	1.00

*Significant at the 5% level.

**Significant at the 1% level.

### Internal Reliability

As shown in Table 11, the Cronbach’s alpha coefficients for the overall TSS and its subscale scores ranged from 0.75 to 0.87, and those for the overall TTS and its subscale scores ranged from 0.74 to 0.90. On the basis of the criterion that a value of 0.70 or higher would be considered acceptable internal reliability (Nunnally, 1978; Nunnally and Bernstein, 1994), we determined that both the TSS and the TSS achieved internal reliability.

**TABLE 11:** Cronbach’s alpha for the TSS and the TTS internal consistency reliability.

	Cronbach’s alpha	No. of items
TSS-F1	0.75	3
TSS-F2	0.87	4
TSS-ALL	0.84	7
TTS-F1	0.74	3
TTS-F2	0.84	5
TTS-F3	0.82	4
TTS-ALL	0.90	12

### Test–Retest Reliability

As summarized in Table 12, the descriptive statistics for both the TSS and the TTS seemed similar for both Time 1 and Time 2. Furthermore, as indicated in Table 13, the Pearson correlation coefficients for the overall TSS and its subscale scores between Time 1 and Time 2 ranged from 0.68 to 0.78, and those for the overall TTS and its subscale scores between the two time points ranged from 0.50 to 0.73. A minimum of 0.70 is needed for acceptable test–retest reliability (Nunnally and Bernstein, 1994). By this standard, the overall TSS and its Factor 1 (Inadequate School-based Support) demonstrated test–retest reliability, while Factor 2 (Teaching-related Demands) was close to achieving test–retest reliability. As for the TTS, its overall scale also demonstrated test–retest reliability, although its three subscales did not.

**TABLE 12:** Descriptive statistics for the TSS and the TTS subscale and overall scale scores at Time 1 (T1, *N* = 122) and Time 2 (T2, *N* = 48).

	Mean (T1)	Mean (T2)	SD (T1)	SD (T2)	Min (T1)	Min (T2)	Max (T1)	Max (T2)
TSS-F1	2.98	2.87	1.03	1.11	1.00	1.00	5.00	5.00
TSS-F2	3.32	3.46	1.02	0.96	1.00	1.50	5.00	4.75
TSS-ALL	3.15	3.17	0.88	0.87	1.13	1.25	4.88	4.71
TTS-F1	2.74	2.81	0.67	0.63	1.00	1.00	4.00	4.00
TTS-F2	2.94	2.88	0.61	0.59	1.00	1.00	4.00	4.00
TTS-F3	2.94	2.94	0.74	0.68	1.00	1.00	4.00	4.00
TTS-ALL	2.85	2.88	0.60	0.54	1.00	1.00	4.00	4.00

*SD, standard deviation; min, minimum; max, maximum.*

**TABLE 13:** Pearson correlation coefficients for the test–retest reliability between the two time points.

		Time 2 (*N* = 48)						
		TSS-F1	TSS-F2	TSS-ALL	TTS-F1	TTS-F2	TTS-F3	TTS-ALL
Time 1 (*N* = 122)	TSS-F1	0.78**						
	TSS-F2		0.68**					
	TSS-ALL			0.76**				
	TTS-F1				0.50**			
	TTS-F2					0.64**		
	TTS-F3						0.66**	
	TTS-ALL							0.73**

**Significant at the 1% level.

## Discussion

Although stress appears to be an ineluctable part of the teaching job, COVID-19 has brought the issue of teacher stress in sharper focus, as teachers’ stress becomes further exacerbated by teaching demands and challenges incurred due to unconventional instructional modalities. Yet, despite intense stress, some teachers are still able to thrive. The question is, how do these teachers develop and sustain their ability to flourish under stressful teaching circumstances? Drawing on theoretical and empirical insights, we developed the TSS and the TTS to shed light on stress and potential thriving dispositions among early childhood teachers during COVID-19. Overall, the findings addressed our research questions as well as confirmed some of our stated hypotheses concerning the validity and reliability of the two scales.

### Validity of the Scales

This study revealed some important findings related to the validity of both the TSS and the TTS. First, both scales seemingly achieved content validity as the scale items were derived from both the theoretical and empirical literature as well as reviewed by early childhood teachers and teacher educators whose feedback was incorporated accordingly in item revisions. This finding suggests the importance of consulting the literature and experts to help enhance content validity. This step was also described as a foundational one in the scale development process (Furr, 2011; Dimitrov, 2012).

Second, we demonstrated that TSS was best represented as multidimensional, suggesting that teacher stress was evoked by two sources: (1) inadequate School-based Support, and (2) Teaching-related Demands. These two factors appeared to have posed as risk factors that engendered teacher stress. It is possible that these two sets of risk factors might have exceeded the protective factors in the teachers’ lives needed to cope with stress constructively (Prilleltensky et al., 2016). This imbalance may be counteracted by increasing social support from multiple sources and reducing teaching-related challenges in multiple areas, a solution that can potentially help mitigate teacher stress.

Third, we originally hypothesized that teacher thriving would consist of six dimensions. However, we only found psychometric evidence to support three latent constructs representing individual qualities with a sufficient number of high-quality items: (1) Adaptability and Flexibility, (2) Personal Strengths and Professional Growth, and (3) Positive Mindset. We did find the existence of other three latent factors regarding teacher thriving in EFA. However, we did not demonstrate a sufficient number of high-quality items to represent these latent constructs. It was a hard decision to exclude those factors but it was more important to maintain the stability and generalizability for the finalized version of the TTS. Thus, we redefined and clarified that our finalized TTS was a measure of teacher thriving with a focus on individual qualities. A common denominator undergirding these factors may be positive changes in personal and professional attributes. This finding aligns with O’Leary and Ickovics’s (1995) and Carver’s (1998) conceptualizations of thriving as involving individual qualities that enable one to flourish. The three sets of individual attributes found in this study also corroborate those reported previously (e.g., hope, Snyder, 1994; optimism, Scheier and Carver, 1992). It is not surprising that these individual attributes contributed to the teachers’ thriving dispositions despite stress because they appeared to be protective resources (Prilleltensky et al., 2016). Furthermore, the psychometric results of the bifactorial nature of teacher stress and the trifactorial nature of teacher thriving suggest that we should define each of these two scales theoretically and practically as multifaceted and dynamic. Thus, any clear understanding or measures of teacher stress and teacher thriving should encompass multiple perspectives.

The fourth finding was that as hypothesized, teacher thriving was negatively correlated with teacher stress. This finding is expected because thriving suggests that individuals are able to overcome stress effectively (Sirois and Hirsch, 2013; Sirois et al., 2017). It is logical that it is only to the extent that a teacher combats stress can he or she flourish. Of the other six constructs (Resilience, Resilience Coping, Coping Efficacy, Teaching Satisfaction, Emotional Support, and Gratitude), Teacher Stress was negatively correlated with Resilience, Coping Efficacy, and Teaching Satisfaction. The negative correlations of teacher stress with resilience and with coping efficacy make sense because individuals who are able to cope with stress more effectively are believed to also be able to become more resilient to stress (Carver, 1998). The finding of the negative correlation between teacher stress and teaching job satisfaction echoes that of previous studies (e.g., Borg et al., 1991; Ho and Au, 2006), and especially that of research concerning early childhood teachers (Carson et al., 2017). These studies all reveal that lower teacher stress tends to be linked to higher teaching job satisfaction, and conversely, higher teacher stress is related to lower teaching job satisfaction.

Fifth, as expected, teacher thriving was found to be associated positively with all of the six convergent constructs. This finding is anticipated because all of these constructs appear to possess protective properties. Protective factors, such as teacher job satisfaction (e.g., Caprara et al., 2006; Ho and Au, 2006), and gratitude (e.g., Chan, 2010, 2011; Howells and Cumming, 2012) can buffer against stress and promote thriving. Furthermore, the finding of the strongest relationship between thriving and coping efficacy is also not surprising. It resonates with Carver’s (1998) conceptualization that thriving individuals are better able to apply efficacious strategies to cope with stress successfully. Thus, it makes sense that in the context of teacher stress, the more able the teachers are in leveraging their efficacious strategies to combat stress, the less stressed and more thriving they will be emotionally.

The positive relationship between stress resilience/resilience coping and teacher thriving found in this study also adds support for O’Leary and Ickovics’s (1995) and Carver’s (1998) conceptualizations attesting to the theoretical linkage between resilience and thriving. Specifically, the finding of teacher thriving, as evident in the teachers’ growth in knowledge, skills, and confidence, also corroborates O’Leary and Ickovics’s (1995) and Carver’s (1998) idea that thriving is similar to but also distinct from resilience in different ways. It then stands to reason that these protective factors are not mutually exclusive assets but operate within a shared buffering system in which opportunities for thriving occur.

### Reliability of the Scales

This study revealed that the overall TSS and the overall TTS and their subscales exhibited good internal consistency reliability. This finding suggests that all of the items within each scale and subscale are interrelated and measured the same construct (Tavakol and Dennick, 2011). This evidence provides support for the scale items being reliable in measuring what they are purported.

This study revealed that the overall TSS and its subscales appeared to demonstrate test–retest reliability. However, the TTS’s subscales did not achieve acceptable test–retest reliability. It may be because the extent of teacher thriving and its three sub-constructs were based on teacher perceptions. Perceptions are considered a mental state-type of measurement that is “highly variable over time” (Price, 2016, p. 228). Although the time length between the test and retest was mostly within the recommended ideal frame (between 14 and 28 days) (Nunnally and Bernstein, 1994), it is plausible that the participating teachers’ interpretations of their thriving capacity might have changed over this time period. However, this lapse of time did not seem to affect the teachers’ reports on the TSS. In fact, the test–retest reliability of the TSS suggests that the teachers’ perceptions of the nature of the stressors appeared to remain similar between the two time frames. Nonetheless, future research might conduct test–retest reliability of measures of both teacher stress and teacher thriving to further confirm or disconfirm the findings reported here.

## Educational and Research Implications

As the world and individual nations continue striving to foster mental health in their citizens, applying the burgeoning knowledge on resilience and building on the emerging understanding of thriving as demonstrated by this study can be instrumental in guiding social policies and actions to promote psychological wellbeing in teachers. Attending to the mental health of early childhood teachers is particularly critical because they are highly prone to stress due to the demanding nature of working with young children (Buettner et al., 2016). In the educational realm, teacher stress presents itself as a pervasive professional turmoil, a phenomenon that potentially erodes human capital. If remained unresolved, teacher stress will likely continue to adversely affect the teachers’ teaching effectiveness and, by extension, the hindrance of student learning and success, especially in early childhood settings (Wells, 2015; Carson et al., 2017; Grant et al., 2019).

To address the aforementioned ramifications, it is imperative that investments in human capital encompass an expansive provision system of support that promotes positive mental health in teachers by ways, such as engaging them in professional development around resilience and thriving discourses. In the short haul, such investments impose time and money. However, in the long haul, they are likely to reap dividends especially by helping to sustain teachers’ professional commitment to teaching and promote their job satisfaction and pedagogical effectiveness, all of which can contribute to a more efficacious teacher workforce to ultimately benefit the educational experiences of students. In this connection, this study aligns with a positive psychology (Seligman and Csikszentmihalyi, 2000) worldview about human functioning by suggesting teacher thriving as a hopeful perspective. Specifically, grounded in the discourse of thriving as a promising mobilizer for potentially re-establishing teaching as a satisfying profession, this study empirically offers teacher thriving as a potentially viable and sustainable mitigation solution to stress, thereby improving the teachers’ mental health needed to potentially achieve teaching efficacy.

Finally, the findings yield theoretical and empirical implications for researchers seeking to better understand thriving as a productive response to any stressful teaching situation confronting teachers. Theoretically, thriving is the optimal response to stress because it promotes personal and professional growth. Empirically, as research on teacher thriving is relatively limited, more efforts are needed to further validate and expand on the theory of teacher thriving by potentially adopting or adapting our new Teacher Thriving Scale and testing it on other teacher populations and even in other teaching contexts.

## Limitations of the Study and Directions for Future Research

This study has conceptual and methodological limitations. We discuss three main ones here. On the conceptual level, although our scale development was informed by theoretical and empirical insights, the items for the TSS and the TTS revolved around only a few selected aspects. Thus, it is possible that we might have missed some key areas. Although it is not practical to create a comprehensive and all-encompassing scale to cover all possible areas, future research might still consider adding other items to the pool to assess other sources of teacher stress and teacher thriving.

On the methodological level, we note a few limitations. First, the small sample size of only 122 participating teachers limits the generalizability of the results. It might also undermine the validity and the reliability of the two newly developed scales. However, the results appear to provide a starting point for understanding the multidimensional dynamics of teacher stress and teacher thriving. Since the TSS and the TTS are brand new and have only been tested in this one study, more research is needed to further evaluate their psychometric properties. Future research testing the TSS and the TTS with larger and more diverse samples might also increase the scales’ generalizability and stability. Relatedly, a second methodological limitation is that we only collected discriminant evidence for construct validity for the TSS. More research testing the TSS with other similar and dissimilar constructs is needed to provide both convergent and discriminant evidence for construct validity.

A third methodological limitation concerns the nature of our sample. Although our full sample consisted of early childhood teachers from 26 states of the United States, it was confined to only members of NAEYC and our teacher acquaintances in New Jersey. This sampling from solely these two means might impose bias and, thus, affect the psychometric results. First, it is possible that those who were willing to participate in both the initial and the follow-up questionnaire were already thriving early childhood teachers. That is, those teachers who first learned about this study via HELLO (the online forum for members of NAEYC) might have already been thriving or seeking social support to thrive through their access to this particular professional network. Second, it is also possible that the participants from New Jersey felt compelled to participate in this study because of their professional relationships with members of the research team. To potentially help mitigate threats to external validity, future research might apply other sampling frames. In this particular case, to further evaluate the reliability and validity of the target scales in the future, researchers might consider conducting a survey through other avenues (e.g., the American Educator Panels, RAND Education and Labor, n.d.) to potentially obtain a larger and more representative national sample across more states of the United States. Furthermore, considering that the levels of teacher stress might vary and opportunities to thrive might be unequally distributed due to varying resources across socio-economic lines of school communities, researchers might conduct stratified sampling of participants across these three types of professional settings (low-income, middle-income, and high-income).

Finally, the research context might also limit the generalizability and stability of the findings. The target scales were tested during a purportedly extra stressful year of teaching due to COVID-19. It is unclear if the psychometric results would still hold true during a different school year amidst COVID-19 or a post-pandemic educational context. To address this contextual limitation, researchers might administer these scales during other times to further evaluate their psychometric properties.

## Data Availability Statement

The raw data supporting the conclusions of this article will be made available by the authors, without undue reservation.

## Ethics Statement

This study involving human participants was reviewed and approved by Kean University’s Institutional Review Board. The participants all indicated online informed consent for participation in this study.

## Author Contributions

JC conceptualized the research design, developed the two new scales, and wrote most of the manuscript. ZL performed all of the statistical analyses as well as wrote the Data Analysis and the Results sections. JC contributed ideas to the writing of these two sections, and ZL to the writing of the Discussion section. WR created and administered the online questionnaire as well as organized the database. JC and SK developed the coding scheme. JC, WR, and SK collected the data. All authors approved the submitted version.

## Conflict of Interest

The authors declare that the research was conducted in the absence of any commercial or financial relationships that could be construed as a potential conflict of interest.

## Publisher’s Note

All claims expressed in this article are solely those of the authors and do not necessarily represent those of their affiliated organizations, or those of the publisher, the editors and the reviewers. Any product that may be evaluated in this article, or claim that may be made by its manufacturer, is not guaranteed or endorsed by the publisher.

## Footnotes

1
www.qualtrics.com

2
https://www.naeyc.org/
